# Systemic chronic inflammation mediates the effect of per- and polyfluoroalkyl substances exposure on the risk of nonalcoholic fatty liver disease: A cross-sectional study among Chinese government employees

**DOI:** 10.1097/EE9.0000000000000411

**Published:** 2025-07-21

**Authors:** Rumeng Xue, Xiuquan Nie, James Ballah, Xiaoqian Du, Xinyu Quan, Qi Li, Minxue Shen, Dan Luo, Shuiyuan Xiao, Yanying Duan

**Affiliations:** a Department of Occupational and Environmental Health, Xiangya School of Public Health, Central South University, Changsha, China; b Hunan Institute for Drug Control, Changsha, China; c Department of Social Medicine and Health Management, Xiangya School of Public Health, Central South University, Changsha, China

**Keywords:** Systemic chronic inflammation, Per- and polyfluoroalkyl substances, Nonalcoholic fatty liver disease, Mediation effect

## Abstract

**Background:**

A series of studies have revealed a correlation between per- and polyfluoroalkyl substances (PFAS) exposure and the increased risk of nonalcoholic fatty liver disease (NAFLD), yet the potential mechanism remains uncertain.

**Objective:**

This study aimed to examine the impact of exposure to PFAS on the risk of developing NAFLD among government employees in China and to investigate the potential mediating role of systemic inflammation.

**Methods:**

This study consisted of 2,191 individuals and the concentration of serum PFAS was determined by ultra-high-performance liquid chromatography-tandem mass spectrometry. Systemic inflammation was assessed by white blood cell count, platelet count, lymphocyte count, neutrophil-to-lymphocyte ratio, and systemic immune-inflammation index. Generalized linear models were used to ascertain the impact of PFAS exposure on the risk of NAFLD. Additionally, a mediation model was applied to determine whether systemic inflammation mediated the association between PFAS exposure and NAFLD risk.

**Results:**

The results indicated that concentrations of perfluorodecanoic acid (odds ratio [OR] = 1.025; 95% confidence interval [CI] = 1.004, 1.046) and perfluorooctane sulfonate (PFOS) (OR = 1.037; 95% CI = 1.014, 1.060) in serum were associated with increased NAFLD risk after adjusting the covariates. The mediation analysis indicated that the estimated 3.50% of the association between PFOS exposure and NAFLD risk was mediated by the systemic immune-inflammation index (*P* = 0.030), which was more prominent among subjects under the age of 40.

**Conclusion:**

Exposure to perfluorodecanoic acid and PFOS may elevate the risk of NAFLD among Chinese government employees, with systemic inflammation serving as a potential mechanism.

**What this study adds**

This study indicated that perfluorodecanoic acid, perfluorooctane sulfonate, and systematic inflammation, could increase the risk of nonalcoholic fatty liver disease (NAFLD), and systemic immune-inflammation index, as a systematic inflammation indicator, mediated the association between perfluorooctane sulfonate exposure and NAFLD risk, which was more prominent among subjects under the age of 40. These findings could contribute to the prevention and control of NAFLD in government employees.

## Introduction

Nonalcoholic fatty liver disease (NAFLD) is one of the well-recognized causes of chronic liver disease on a global scale and has become an increasingly prominent public health concern.^1,2^ Epidemiological findings indicated that the prevalence of NAFLD in Asia reached 30%, comparable to that of European countries.^3^ The total number of cases of NAFLD in China is predicted to reach 314,580,000 in 2030.^4^ Recently, great attention has been paid to certain environmental pollutants, in addition to other traditional risk factors such as diet, lifestyle, and genetics in exploring the potential pathogenic factors of NAFLD.^5^

Per- and polyfluoroalkyl substances (PFAS) constitute a class of persistent, hazardous chemicals with unique physicochemical properties, including perfluorooctanoic acid (PFOA), perfluorononanoate (PFNA), perfluorodecanoic acid (PFDA), perfluorohexane sulfonic acid (PFHxS), and perfluorooctane sulfonate (PFOS).^6^ Human exposure to PFAS can occur through inhalation of dust, ingestion of water and food, and direct dermal contact, and as a result, PFAS can be found in a wide range of human tissue or organs,^7,8^ including liver samples.^9^ A systematic review indicated that PFAS have hepatotoxic effects and are associated with an enhanced risk of hepatocellular carcinoma.^10^ They can disrupt hepatic lipid metabolism and induce hepatic steatosis and fibrosis in both in vivo and in vitro experiments.^11,12^ Based on the data from the National Health and Nutrition Examination Survey (NHANES), PFOA and PFNA were significantly associated with NAFLD risk in various regression model.^13–15^ Notably, PFOS and PFHxS were linked to the histological severity of NAFLD in children.^14^ However, the potential mechanism underlying the adverse influence of PFAS exposure on NAFLD risk remains elusive.

It is widely acknowledged that exposure to PFAS could initiate a pro-inflammatory state. A study found an increased trend of serum C-reactive protein when explored the penetration ability of PFAS across blood cerebrospinal.^16^ Based on the NHANES database (2005–2012), PFOA, PFOS, PFNA, PFHxS, and PFDA were found to be positively associated with lymphocyte (LY) count,^17^ which was further confirmed by another investigation conducted in a PFOA drinking water contamination district of USA.^18^ Consistently, increased PFAS exposure in pregnant women was related to increases in interferon-γ, interleukin 10 (IL-10), and tumor necrosis factor α (TNF-α).^19^ A recently published review suggested that the ability to induce chronic inflammation by PFAS probably contributed to the pathogenesis of most adverse health effects.^20^ Notably, inflammation was a prominent contributor to the occurrence and progression of NAFLD,^21^ and most of the intervention strategy, including lifestyle modification and pharmacological treatment, was unexceptionally focused on the block of inflammation.^22^ Currently, animal experiments have revealed that the application of PFAS led to the activation of peroxisome proliferator-activated receptor α (PPARα), which is primarily involved in inflammation and fatty acid metabolism and lipid homeostasis.^23^ Therefore, it is plausible that systemic inflammation probably be engaged in the process of NAFLD occurrence among subjects with high PFAS exposure.

White blood cell (WBC), platelet (PLT), and LY are crucial cellular components that are linked to immunity and inflammation. In recent decades, some comprehensive indices, specifically the neutrophil-to-lymphocyte ratio (NLR) and systemic immune-inflammatory index (SII), have emerged as cost-effective biological markers that manifest the balance between systemic inflammation and immunity, and are widely used in clinical practice.^24^ They have also been applied in the prediction of the deterioration of NAFLD.^25,26^ Recently, SII levels exhibit a positive association with the degree of hepatic steatosis,^27^ and U-shaped correlated with the risk of NAFLD.^28^ Nevertheless, to the best of our knowledge, it remains unclear whether these inflammation indicators mediate the association between PFAS exposure and NAFLD risk.

To fill the knowledge gap, we carried out a cross-sectional study among governmental employees. After estimating the levels of PFAS in serum samples, we first explored the pairwise association among PFAS exposure, inflammation indicators, and NAFLD risk, and then evaluated the potential mediating role of systemic chronic inflammation, indicated by WBC, PLT, LY, NLR, and SII.

## Methods

### Study population

All the participants were from the cohort study among government employees in Hunan Province, China, which aimed to explore the risk factor for chronic disease and was described elsewhere.^29^ Briefly, subjects in this study were enrolled from January 2018 to October 2019 and invited to take part in an investigation containing physical examination, questionnaires, and laboratory tests at the Health Management Center of the Third Xiangya Hospital in Changsha City. Both questionnaires and physical examinations were performed by trained investigators. All the participants were divided into different subgroups based on gender and age (with a range of 10 years old). Also, 2,566 individuals were selected from the total population by random sampling according to the proportion of subgroups in the total population. Among them, 192 participants with excess alcohol intake (>70 g/week for women; >140 g/week for men),^30^ 17 subjects with a history of chronic hepatitis, cirrhosis, or liver cancer, 163 subjects with missing basic information, and three participants with missing hematological parameter data were excluded, and at last, a total of 2,191 employees were included in the following study. Written informed consent was received from all participants before the survey. The study was approved by the institutional review board of Xiangya School of Public Health, Central South University (No.XYGW–2016–10).

### Exposure assessment and laboratory analyses

Eight kinds of PFAS, including PFOA, PFNA, PFDA, PFHxS, PFOS, perfluorobutanoic acid, perfluoroheptanoic acid, and perfluoroundecanoic acid (PFUnDA) were quantified in serum samples, based on a method described by other investigators.^31^ In short, after thawing, each sample (150 μL) was added to precooled extraction solution (450 μL) and vortexed for 30 seconds. They were subjected to sonication in an ice-water bath for 20 minutes and subsequently centrifuged (15 minutes, 12,000 rpm, 4°C). The clear supernatants were subjected to analysis using ultra-high-performance liquid chromatography-tandem mass spectrometry (Agilent 1290 UHPLC-6460 MS). The SCIEX Analyst Work Station Software (Version 1.6.3) and Multiquant 3.03 software were utilized for the acquisition and processing of MRM data. The following analysis is limited to those PFAS with a detectable rate >90% in the samples: PFOA, PFNA, PFDA, PFUnDA, PFHxS, and PFOS.

### Assessment of systemic inflammation

Blood samples with anticoagulant (5 mL) from each subject were centrifuged and the supernatants were collected. Testing was completed within 5 hours. Hematological parameters, including WBC, PLT, NLR, and LY counts, were obtained by using an automatic flow cytometer (Beckman, California) with reagents supplied by the same company, strictly following the instructions. Comprehensive indicators associated with systemic immune inflammation were calculated: NLR = NEU/LY, SII = PLT × NEU/LY, where PLT, NEU, and LY represented the counts of PLT, neutrophils, and LYs, respectively. In this study, WBC, PLT, LY, NLR, and SII were applied to evaluate the degree of systemic inflammation response.

### Diagnosis of NAFLD

NAFLD is a clinicopathological syndrome characterized by diffuse macrovesicular steatosis and includes simple fatty liver and nonalcoholic steatohepatitis and cirrhosis that evolve from it. After exclusion of the subjects with excessive drinking, viral hepatitis, and drug-induced liver disease, participants would be considered NAFLD patients if they exhibited symptoms of metabolic syndrome with increased transaminase and abnormal ultrasound examination results. The detailed diagnostic criteria were referenced from the Chinese Guidelines for the diagnosis and treatment of NAFLD, which was described by other investigator.^32^

### Covariates

Age (years), gender (male/female), marriage (unmarried, married, divorced, or widowed, or other), family income (CNY) (<50,000, 50,000–100,000, 110,000–200,000, 210,000–300,000, 310,000–500,000, 510,000–1,000,000, or >1,000,000), body mass index (BMI) (kg/m^2^), physical activity (yes/no), sedentary time (<2 hours, 2–4 hours, 4–6 hours, or >6 hours), smoking (never, former smoker, or currently smoking), drinking (never, former drinker, or currently drinking), hypertension (yes/no), and diabetes (yes/no) were included in the analyses. BMI was obtained by dividing weight by the square of height. For physical activity, regular status was defined as more than 1 day per week. Those participants who reported smoking at least one cigarette per day for a period exceeding 6 months were classified as current smokers, while those who had ceased smoking more than 6 months prior were considered former smokers.^29^ Similarly, participants who consumed alcohol at least once a week for a period exceeding 6 months were classified as current drinkers, while those who had quit drinking more than 6 months prior were considered former drinkers.^33^ Hypertension was defined as systolic blood pressure more than or equal to 140 mm Hg, diastolic blood pressure more than or equal to 90 mm Hg, and/or treatment with antihypertensive medication.^34^ Diabetes was defined as a fasting glucose level of more than or equal to 7.0 mmol/L or treatment with antidiabetic medication.^35^

### Statistical analysis

Continuous variables exhibiting normal distribution were described as mean ± standard deviation (SD) while those without normal distribution were expressed as medians along with interquartile range (IQR), which were compared by analysis of variance and Wilcoxon rank-sum test, respectively. For categorical variables, they were described as numbers and percentages, and evaluated by applying the *χ*^*2*^ test. Concentrations of PFAS and systemic inflammatory indicators were natural logarithm(ln)-transformed in subsequent analyses due to the skewed distributions. The correlations of environmental chemicals were assessed by calculating Spearman’s rank correlation coefficient, which was presented as a heat map. Generalized linear model was applied to explore the relationship between PFAS and systemic inflammatory markers, which generated regression coefficient (*β*) and 95% confidence intervals (95% CI). Participants who self-reported or were diagnosed as NAFLD via abdominal ultrasound were classified into the NAFLD group and the rest subjects were classified into the control group. Generalized linear model was employed to analyze the association of NAFLD with PFAS exposure or systemic inflammation indicators. To gain insight into the mechanism through which PFAS exposure affects NAFLD risk, we conducted a mediation analysis of systemic inflammation. PFDA and PFOS were selected as independent variables due to their significant associations with NAFLD. Whether NAFLD occurred was used as the outcome variable. The mediation effect analysis was carried out by using the “Mediation” package in R software. In addition, we stratified participants by gender (male/female), age (<40 years old/≥40 years old), BMI (<24 kg/m²/≥24 kg/m²), and physical activity (yes/no) to explore the characteristics of potentially sensitive populations. Age, gender, marriage, family income, BMI, physical activity, sedentary time, smoking, drinking, hypertension, and diabetes were adjusted in all models. Notably, NAFLD included the diagnosis of nonalcoholic steatohepatitis, and inflammation can also be a result of NAFLD. The cross-sectional design of this investigation could not exclude the potential mediation effect of NAFLD between PFAS exposure and inflammation. Therefore, we further performed a bidirectional mediation analysis.

All analyses were conducted using R version 4.3.1 and SPSS version 26.0 (SPSS Inc., Chicago, Illinois). All statistical tests were 2-sided, and differences were considered statistically significant with *P* < 0.05.

## RESULTS

### Participant characteristics

The basic information of 2,191 individuals was summarized in Table 1. The mean and SD of age were 39.7 and 9.2 years old, respectively. Almost 51.1% of participants were women. Compared to subjects without NAFLD, participants with NAFLD tended to be older, male, and had higher BMI. All basic characteristics, except physical activity, differed significantly between NAFLD and non-NAFLD groups with *P* < 0.05.

**Table 1.** Characteristics of the participants categorized by NAFLD status

	All (n = 2,191)	NAFLD (n = 658)	Non-NAFLD (n = 1,533)	*P*
Basic information				
Age, years, mean (SD)	39.7 (9.2)	41.9 (8.8)	38.8 (9.2)	<0.001
Gender, n (%)				
Male	1093 (49.9)	524 (79.6)	569 (37.1)	<0.001
Female	1098 (50.1)	134 (20.4)	964 (62.9)	
Marriage, n (%)				
Unmarried	251 (11.5)	43 (6.5)	208 (13.6)	<0.001
Married	1879 (85.8)	602 (91.5)	1277 (83.3)	
Divorced or widowed	54 (2.5)	11 (1.7)	43 (2.8)	
Other	7 (0.3)	2 (0.3)	5 (0.3)	
Family income (CNY), n (%)				
<50,000	216 (9.9)	65 (9.9)	151 (9.8)	0.027
50,000–100,000	499 (22.8)	134 (20.4)	365 (23.8)	
110,000–200,000	841 (38.4)	245 (37.2)	596 (38.9)	
210,000–300,000	422 (19.3)	139 (21.1)	283 (18.5)	
310,000–500,000	167 (7.6)	58 (8.8)	109 (7.1)	
510,000–1,000,000	34 (1.6)	11 (1.7)	23 (1.5)	
>1,000,000	12 (0.5)	6 (0.9)	6 (0.4)	
BMI, kg/m², median (IQR)	23.23 (21.22, 25.68)	25.89 (24.22, 27.64)	22.20 (20.50, 24.00)	<0.001
Physical activity, n (%)				
Yes	1315 (60.0)	415 (63.1)	900 (58.7)	0.056
No	876 (40.0)	243 (36.9)	633 (41.3)	
Sedentary time, n (%)				
<2 hours	553 (25.2)	140 (21.3)	413 (26.9)	<0.001
2–4 hours	980 (44.7)	287 (43.6)	693 (45.2)	
4–6 hours	408 (18.6)	150 (22.8)	258 (16.8)	
>6 hours	250 (11.4)	81 (12.3)	169 (11.0)	
Smoking, n (%)				
Never	1828 (83.4)	473 (71.9)	1355 (88.4)	<0.001
Former	53 (2.4)	26 (4.0)	27 (1.8)	
Current	310 (14.1)	159 (24.2)	151 (9.8)	
Drinking, n (%)				
Never	1884 (86.0)	496 (75.4)	1388 (90.5)	<0.001
Former	21 (1.0)	13 (2.0)	8 (0.5)	
Current	286 (13.1)	149 (22.6)	137 (8.9)	
Hypertension, n (%)				
No	2150 (98.1)	634 (96.4)	1516 (98.9)	<0.001
Yes	41 (1.9)	24 (3.6)	17 (1.1)	
Diabetes, n (%)				
No	2159 (98.5)	638 (97.0)	1521 (99.2)	<0.001
Yes	32 (1.5)	20 (3.0)	12 (0.8)	
Serum concentrations of PFAS (ng/mL), median (IQR)				
PFOA	19.4 (10.1, 29.5)	19.9 (9.8, 29.9)	19.2 (10.3, 29.3)	0.750
PFNA	10.8 (5.4, 16.1)	10.8 (5.5, 16.4)	10.8 (5.4, 16.1)	0.937
PFDA	4.4 (2.3, 6.9)	4.8 (2.7, 8.1)	4.2 (2.2, 6.4)	<0.001
PFUnDA	3.9 (2.2, 6.8)	3.8 (2.3, 6.3)	4.1 (2.1, 7.0)	0.214
PFHxS	2.6 (1.4, 3.8)	2.6 (1.3, 3.8)	2.7 (1.4, 3.8)	0.591
PFOS	14.9 (8.6, 21.2)	16.2 (9.5, 23.3)	14.2 (8.3, 20.8)	<0.001
ΣPFAS	59.1 (47.2, 72.7)	61.6 (49.1, 75.4)	58.4 (46.4, 71.4)	0.002
Systemic inflammation indicators, median (IQR)				
WBC, ×10^9^/L	6.13 (5.17, 7.05)	6.5 (5.66, 7.56)	5.9 (4.99, 6.87)	0.001
PLT, ×10^9^/L	219.00 (188.00, 253.00)	222.00 (189.00, 255.25)	217.00 (188.00, 251.00)	0.128
LY, ×10^9^/L	1.99 (1.66, 2.37)	2.14 (1.76, 2.56)	1.92 (1.62, 2.28)	<0.001
NLR	1.76 (1.39, 2.24)	1.78 (1.41, 2.23)	1.75 (1.39, 2.24)	0.525
SII	377.83 (287.80, 512.06)	389.40 (291.82, 503.77)	371.57 (283.99, 514.82)	0.203

Association between PFAS exposure and NAFLD risk among Chinese government employees.

The medians of PFAS (ng/mL) among total population were 19.40 for PFOA, 10.80 for PFNA, 4.40 for PFDA, 3.90 for PFUnDA, 2.60 for PFHxS, and 14.90 for PFOS, respectively. The serum levels of PFDA and PFOS differed significantly between the NAFLD group and the non-NAFLD group. In terms of systemic inflammation indicators, the medians of WBC, PLT, and LY were 6.13 × 10^9^/L, 219.00 × 10^9^/L, and 1.99 × 10^9^/L, respectively and the medians of NLR and SII were 1.76 and 377.83. The NAFLD group demonstrated significantly higher concentrations of the WBC (*P* < 0.001) and LY (*P* < 0.001) when compared to the non-NAFLD group. Figure S1 and Table S1; https://links.lww.com/EE/A360 displayed the results of correlation analysis among six types of PFAS and five kinds of immune-inflammation indicators, with correlation coefficient ranging from −0.53 to 0.84.

**Figure 1.**
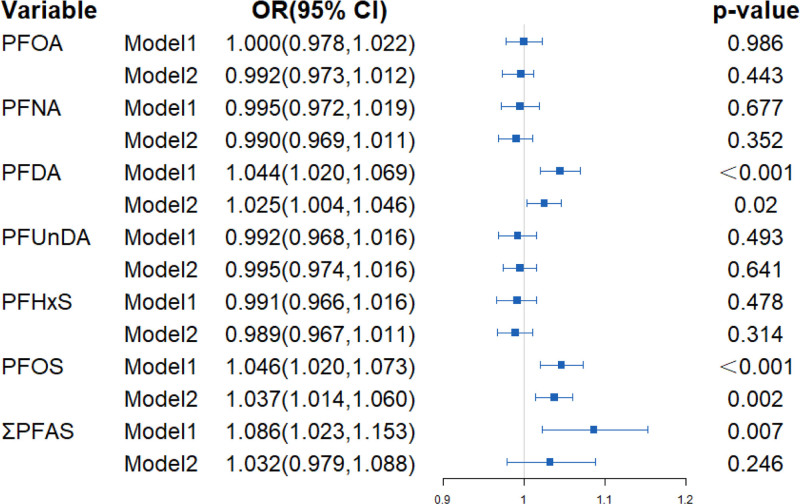
Association between PFAS exposure and NAFLD risk analyzed by generalized linear models. The results were presented as odds ratios (OR) and 95% confidence interval (95% CI). Model 1 is an unadjusted crude model, while Model 2 is adjusted for age, gender, marriage, family income, BMI, physical activity, sedentary time, smoking, drinking, hypertension, and diabetes.

As shown in Figure 1, participants with higher concentrations of PFDA (odds ratio [OR] = 1.025; 95% confidence interval [CI]: 1.004, 1.046) and PFOS (OR = 1.037; 95% CI = 1.014, 1.060) had significantly increased risks of NAFLD after adjustment of age, gender, marriage, family income, BMI, physical activity, sedentary time, smoking, drinking, hypertension, and diabetes mellitus.

### Association between PFAS exposure and systemic inflammation analyzed by generalized linear model

**Figure 2.**
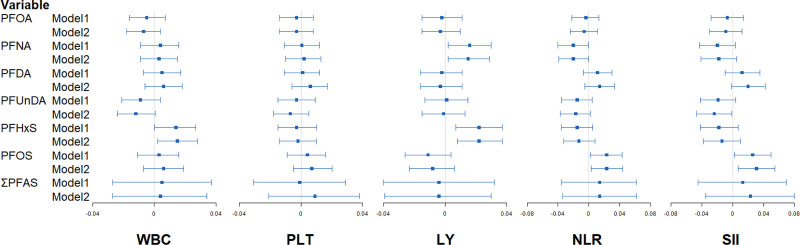
Forest plot of the association between PFAS exposure and systemic inflammation indicators analyzed by generalized linear models. Model 1 is an unadjusted crude model, while Model 2 is adjusted for age, gender, marriage, family income, BMI, physical activity, sedentary time, smoking, drinking, hypertension, and diabetes.

In Figure 2 and Table S2; https://links.lww.com/EE/A360 the results revealed that PFOS was positively correlated with NLR (*β* = 0.023; 95% CI = 0.003, 0.044) and SII (*β* = 0.031; 95% CI = 0.007, 0.055) after fully adjusting for the covariates. In addition, higher levels of PFHxS showed significant associations with higher levels of WBC (*β* = 0.015; 95% CI = 0.002, 0.028) and LY (*β* = 0.022; 95% CI = 0.008, 0.037). PFNA was positively correlated with LY (*β* = 0.015; 95% CI = 0.002, 0.029), however, negatively correlated with NLR (*β* = −0.020; 95% CI = −0.039, −0.001). PFUnDA was negatively correlated with SII (*β* = −0.024; 95% CI = −0.047, −0.001).

### Association between systemic inflammation and NAFLD risk among Chinese government employees

**Figure 3.**
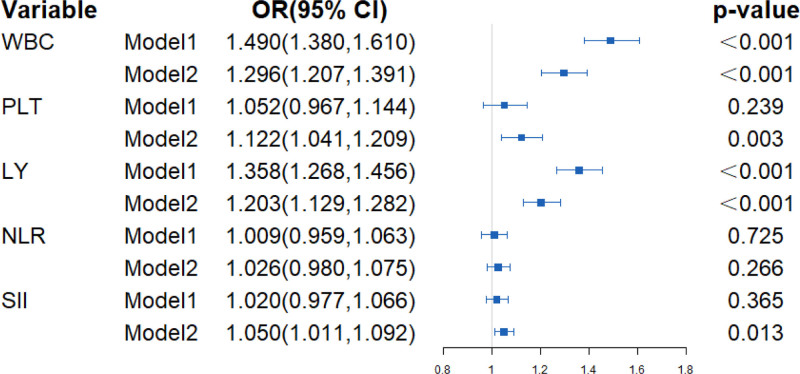
Association between systemic inflammation indicators and NAFLD risk analyzed by generalized linear models. The results were presented as odds ratios (OR) and 95% confidence interval (CI). Model 1 is an unadjusted crude model, while Model 2 is adjusted for age, gender, marriage, family income, BMI, physical activity, sedentary time, smoking, drinking, hypertension, and diabetes.

A statistically positive association was found for NAFLD risk with WBC, PLT, LY, and SII, as Figure 3 indicated. For one unit increase in WBC, PLT, LY, and SII, the NAFLD risk was 1.296 (95% CI = 1.207, 1.391), 1.122 (95% CI = 1.041, 1.209), 1.203 (95% CI = 1.129, 1.282) and 1.050 (95% CI = 1.011, 1.092) times higher than the reference.

### Mediation effect analysis of systemic inflammation on the association between PFAS exposure and NAFLD risk

In the mediation effect model, PFDA and PFOS levels, NAFLD risk, and systemic inflammation indicators were applied as independent variable, dependent variable, and mediating variable, respectively. As indicated in Table 2 (adjusted model), the mediated effect of SII accounted for 3.50% of the total effect of PFOS exposure and NAFLD risk after fully adjusting for the covariates (*P* = 0.030), however, which disappeared in the crude model (Table S3; https://links.lww.com/EE/A360). The result in Table S4; https://links.lww.com/EE/A360 failed to find a mediation effect of NAFLD between PFAS exposure and inflammatory indicators.

**Table 2.** Mediation effect of systemic inflammatory indicators in the association between PFAS exposure and NAFLD risk^a^

Parameters	Direct effect	Indirect effect	Total effect	Percentage mediated (%)	*P*-mediated
PFDA-NAFLD					
WBC	**0.023 (0.001, 0.043**)^c^	0.001 (−0.002, 0.004)	**0.024 (0.003, 0.044**)^c^	5.58	0.414
PLT	**0.023 (0.002, 0.044**)^c^	0.001 (−0.001, 0.002)	**0.024 (0.002, 0.044**)^d^	2.26	0.368
LY	**0.024 (0.003, 0.045**)^d^	−0.001 (−0.003, 0.002)	**0.024 (0.002, 0.044**)^c^	-^b^	-
NLR	**0.024 (0.002, 0.044**)^c^	0.0003 (−0.0003, 0.001)	**0.024 (0.002, 0.044**)^c^	1.00	0.404
SII	**0.023 (0.002, 0.044**)^c^	0.001 (−0.0002, 0.002)	**0.024 (0.002, 0.044**)^d^	3.19	0.136
PFOS-NAFLD					
WBC	**0.034 (0.012, 0.055**)^e^	0.001 (−0.002, 0.005)	**0.035 (0.013, 0.057**)^e^	3.70	0.454
PLT	**0.035 (0.012, 0.057**)^e^	0.001 (−0.001, 0.002)	**0.035 (0.014, 0.057**)^e^	1.95	0.284
LY	**0.037 (0.015, 0.058**)^e^	−0.002 (−0.005, 0.001)	**0.035 (0.014, 0.056**)^e^	-	-
NLR	**0.035 (0.012, 0.056**)^e^	0.001 (−0.0005, 0.002)	**0.035 (0.013, 0.056**)^e^	1.21	0.320
SII	**0.034 (0.012, 0.056**)^e^	**0.001 (0.0001, 0.003**)^c^	**0.035 (0.013, 0.057**)^e^	**3.50**	**0.030** ^c^
ΣPFAS-NAFLD					
WBC	0.028 (−0.025, 0.081)	0.001 (−0.007, 0.009)	0.029 (−0.024, 0.082)	2.67	0.814
PLT	0.029 (−0.027, 0.082)	0.001 (−0.002, 0.005)	0.030 (−0.026, 0.083)	1.72	0.670
LY	0.030 (−0.024, 0.082)	−0.001 (−0.007, 0.006)	0.029 (−0.024, 0.082)	-	-
NLR	0.02 9 (−0.026, 0.082)	0.0004 (−0.001, 0.002)	0.029 (−0.024, 0.082)	0.45	0.700
SII	0.02 8 (−0.027, 0.083)	0.001 (−0.002, 0.005)	0.029 (−0.026, 0.083)	2.06	0.600

The results displayed in bold indicate statistical significance.

a The results were adjusted for age, gender, marriage, family income, BMI, physical activity, sedentary time, smoking, drinking, hypertension, and diabetes.

b The mediation ratio cannot be estimated because the direct and indirect effects are in opposite directions.

c *P <* 0.05.

d *P <* 0.01.

e *P <* 0.001.

### Stratification analysis

After stratified by gender, BMI, and physical activity, the mediation effect of SII between PFAS and NAFLD remained among participants under the age of 40 with an estimated percentage of 7.90% (*P* = 0.058), as shown in Table 3.

**Table 3.** Stratification analysis of the mediation effect of SII in the association between PFOS exposure and NAFLD risk^a^

Parameters	N	Direct effect	Indirect effect	Total effect	Percentage mediated(%)	*P*-mediated
Gender						
Male	1093	0.030 (−0.007, 0.064)	0.002 (−0.0005, 0.006)	0.032 (−0.005, 0.068)	5.52	0.224
Female	1098	**0.045 (0.022, 0.067**)^c^	0.001 (−0.001, 0.003)	**0.046 (0.022, 0.068**)^c^	1.30	0.340
Age, years						
<40	1186	**0.031 (0.002, 0.058**)^d^	**0.003 (0.0002, 0.007**)^d^	**0.034 (0.005, 0.062**)^d^	**7.90**	**0.058**
≥40	1005	**0.038 (0.004, 0.071**)^d^	−0.0003 (−0.002, 0.001)	**0.038 (0.004, 0.071**)^d^	-^b^	-
BMI, kg/m²						
<24	1287	**0.045 (0.025, 0.063**)^c^	0.001 (−0.0003, 0.003)	**0.045 (0.026, 0.063**)^c^	1.57	0.170
≥24	904	0.021 (−0.021, 0.061)	0.001 (−0.001, 0.004)	0.022 (−0.021, 0.063)	2.11	0.610
Physical activity						
Yes	1315	**0.032 (0.002, 0.060**)^d^	0.001 (−0.001, 0.003)	**0.032 (0.002, 0.061**)^d^	1.79	0.400
No	876	**0.041 (0.007, 0.072**)^d^	0.002 (−0.001, 0.005)	**0.043 (0.010, 0.075**)^d^	3.36	0.280

The results displayed in bold indicate statistical significance.

a The results were adjusted for age, gender, marriage, family income, BMI, physical activity, sedentary time, smoking, drinking, hypertension, and diabetes.

b The mediation percentage cannot be estimated because the direct and indirect effects are in opposite directions.

c *P <* 0.001.

d *P <* 0.05

## DISCUSSION

Our results indicated that PFDA and PFOS were positively associated with the risk of NAFLD. There are positive correlations of PFHxS with WBC and LY, PFOS with NLR, and SII and PFNA with LY. All the inflammatory indicators were positively associated with the risk of NAFLD except NLR. Most importantly, SII was found to be involved in the association between PFOS exposure and NAFLD risk and played an estimated mediating role of 3.50%, which remained significant among participants under the age of 40. To the best of our knowledge, this is the first population-based study reporting the mediation effect of systemic inflammation on the association between PFAS exposure and NAFLD risk.

The hepatotoxicity of PFAS has been widely reported in human and rodent research, mainly manifested as atypical steatosis, hepatomegaly, and hepatocarcinogenesis.^10^ Based on three cycles of the NHANES database, PFDA was reported to be positively associated with NAFLD risk when it was in a quartile form, and multivariate linear regression demonstrated a positive association between serum PFOS concentration and elevated hepatic steatosis index.^36^ Also, based on the NHANES database, Zhang and colleagues found that PFAS exposure was more closely associated with hepatic fibrosis than steatosis, and PFOS might be the prominent component.^37^ In line with these results, our study also indicated that NAFLD risk was significantly higher among subjects with higher PFOS and PFDA levels. However, the epidemiological evidence on the relationship between PFOS level and NAFLD risk remained inconclusive. A case–control study in northwest China identified a nonlinear negative association between PFOS exposure and the risk of developing NAFLD.^38^ The discrepancy was probably attributable to the difference in exposure level, with a PFOS median of 2.42 μg/L in this study, more than 6 times lower than that in our study (14.9 μg/L). In addition, PFAS exposure duration, personal lifestyle, dietary habits, and the statistical model were all potential influential factors.

Inflammation is a common stress response to PFAS exposure in multiple tissues and organs.^39^ PFOS treatment could initiate significant accumulations of neutrophils and macrophages and promote the infiltration of inflammatory cytokines.^40^ An animal experiment also found that high exposure to PFOS altered LY proliferation.^41^ In population-based research, NLR and SII are novel inflammatory markers that integrate PLTs, NLRs, and LYs, and can comprehensively reflect the magnitude of inflammation in the human body.^42^ In this study, our results provided additional evidence that the levels of NLR, SII, LY, and WBC significantly increased when exposed to PFAS, which was partly in line with the outcome of NHANES.^17^ Notably, systemic inflammation is implicated in the development and progression of NAFLD. A systematic review has indicated that NLR may serve as an independent prognostic marker for liver fibrosis in patients with NAFLD.^26^ Furthermore, Xie et al^43^ found that SII is an independent risk factor for hepatic steatosis for the first time based on the 2017–2020 US NHANES dataset. These reports supported our findings that SII, WBC, PLT, and LY were all risk factors for NAFLD and also positively associated with PFAS exposure.

The potential molecular mechanisms underlying the influences of PFDA and PFOS on NAFLD risk in the human body remain unclear. For the first time, our results indicated that systemic inflammation probably mediated the adverse effect based on population investigation. Presently, evidence from rodent experiment has found that administration of PFOS can activate NLRP3 inflammasome, thereby triggering hepatic steatosis through induction of significant inflammatory injury.^44^ As a ligand-activated nuclear receptor, PPARα regulated the pro-inflammatory signaling pathway in acute-phase response to external pollutants.^45^ A cross-sectional study identified PPARα activation as a key molecular event in the dysregulation of hepatic lipid accumulation induced by PFOS in rodent model and human investigation.^36^ Furthermore, proteomics research revealed that after exposure to PFOS during pregnancy and lactation, the most significantly differentially expressed proteins were Acox1, Acot1, and Acot2, which were all involved in lipolytic metabolism, lipid synthesis, hepatic inflammation, and steatosis.^46^ It is worth noting that the mediation percentage of systematic inflammation in this study is limited to 3.50%, suggesting the potential involvement of other biological mechanisms related to NAFLD occurrence after PFAS exposure. Due to the structural similarity with a fatty acid, PFAS could bind to human liver fatty acid binding protein and interfere with the absorption, transportation, and storage processes,^47^ which would lead to abnormal accumulation of fatty acid in the liver. In liver cell models, it was found that PFAS treatment triggered the production of endogenous reactive oxygen species and activated unfolded protein response, accompanied by increases in steatosis and fibrosis biomarkers.^12^ It is plausible that oxidative stress and the subsequent cascade reaction probably contribute to the pathophysiological change of NAFLD. In addition, the interference with energy metabolism by PFAS probably exerted a stable effect on the development of NAFLD between generations. For example, Blake et al^48^ observed that peroxisome, oxidative phosphorylation, adipogenesis, and bile acid metabolism were the shared biological process that was related to the liver toxicity for PFAS exposure in both maternal and fetal livers.

The results of the subgroup analyses indicated an age-related discrepancy in the mediating effect of SII on the increased risk of NAFLD due to PFOS exposure. Consistently, Hu et al^25^ reported that the positive association of SII level and metabolic syndrome risk remained significant in individuals with age less than 39 years based on the data from NHANES 2011–2016, and they speculated that it was probably due to the declines in immune cell function and inflammatory response intensity among old people.^49^ It is possible that a nonsystemic inflammation-mediated mechanism may be responsible for the increased risk of NAFLD associated with PFOS exposure among old people.

Our study also had several limitations. First, multiple testing in our regression analyses would increase the likelihood of chance findings, especially for those results with marginal significance. Second, the pathophysiological change of NAFLD was a long-term process. However, the levels of systemic inflammatory markers and PFAS concentration, only measured once in this study, could not accurately reflect the status and exposure level over a long period. Due to the cross-sectional design, the mediation effect of SII on the association between PFAS exposure and NAFLD risk should be explained with caution. In addition, it should be noted that our findings were not representative of the general population, as the participants were limited to government employees, who were likely to have longer sedentary time and higher education levels than other people. Therefore, further longitudinal study with repeated measurement was warranted to validate the result in this study.

## Conclusions

In conclusion, our findings indicated that PFDA,d PFOS, and systematic inflammation could increase the risk of NAFLD, and SII, as a systematic inflammation indicator, medicated the association between PFOS exposure and NAFLD risk, which was more prominent among subjects under the age of 40. These findings could contribute to the prevention and control of NAFLD in government employees. However, further studies are warranted to confirm the findings.

## Acknowledgments

We are grateful to all the participants in this study, the interviewers who supported data collection, and the physicians who gave advice on physical examination. We are also grateful to anonymous reviewers and the editor for their suggestions and comments.

## Conflicts of interest statement

The authors declare that they have no conflicts of interest with regard to the content of this report.

## Supplementary Material
