# Supplementary material for: Systemic chronic inflammation mediates the effect of per- and polyfluoroalkyl substances exposure on the risk of nonalcoholic fatty liver disease: A cross-sectional study among Chinese government employees
Source: Environ Epidemiol. 2025 Jul 21;9(4):e411. doi: 10.1097/EE9.0000000000000411 (PMC12282732; doi:10.1097/EE9.0000000000000411)
Supplement: Supplementary file 1 [file ee9-9-e411-s001.pdf]

Supplementary Materials

Table S1. Spearman correlation matrix of PFAS and systemic inflammation indicators among the total population (n = 2191).

	PFOA	PFUnDA	PFNA	LY	PFHxS	WBC	PLT	SII	NLR	PFDA	PFOS
PFOA	1.00										
PFUnDA	0.02	1.00									
PFNA	−0.03	**−0.06***	1.00								
LY	−0.01	0.00	**0.04***	1.00							
PFHxS	−0.02	−0.04	**0.05***	**0.07****	1.00						
WBC	0.00	−0.03	0.03	**0.58*****	**0.05***	1.00					
PLT	−0.01	0.00	0.00	**0.22*****	0.00	**0.28*****	1.00				
SII	0.00	−0.04	−0.03	**−0.32*****	−0.03	**0.42*****	**0.53*****	1.00			
NLR	0.00	**−0.04***	−0.04	**−0.53*****	−0.03	**0.32*****	0.04	**0.84*****	1.00		
PFDA	−0.01	**−0.22*****	**0.05***	−0.02	0.04	0.01	−0.01	0.03	0.03	1.00	
PFOS	0.03	**−0.22*****	**0.07*****	−0.04	**0.06****	0.00	0.00	**0.05***	**0.06****	**0.26*****	1.00

* $p < 0.05$, ** $p < 0.01$ and *** $p < 0.001$.

Table S2. Association of single PFAS exposure levels with systemic inflammation indicators based on generalized linear models.

		WBC		PLT		LY		NLR		SII	
		β (95% *CI*)	*p*-value	*β* (95% *CI*)	*p*-value	*β* (95% *CI*)	*p*-value	*β* (95% *CI*)	*p*-value	*β* (95% *CI*)	*p*-value
PFOA	Model 1	−0.005(−0.016,0.007)	0.418	−0.003(−0.014,0.008)	0.605	−0.002(−0.015,0.011)	0.747	−0.004(−0.022,0.013)	0.627	−0.007(−0.028,0.014)	0.499
	Model 2	−0.007(−0.018,0.004)	0.233	−0.003(−0.014,0.008)	0.608	−0.003(−0.015,0.010)	0.665	−0.006(−0.024,0.011)	0.486	−0.009(−0.030,0.012)	0.394
PFNA	Model 1	0.004(−0.009,0.016)	0.584	0.0005(−0.011,0.012)	0.940	**0.016(0.002,0.030)**	**0.026**	**−0.020(−0.040,−0.001)**	**0.037**	−0.020(−0.043,0.003)	0.087
	Model 2	0.003(−0.009,0.015)	0.623	0.002(−0.010,0.013)	0.786	**0.015(0.002,0.029)**	**0.028**	**−0.020(−0.039,−0.001)**	**0.043**	−0.018(−0.041,0.005)	0.117
PFDA	Model 1	0.005(−0.007,0.017)	0.413	0.001(−0.011,0.012)	0.899	−0.002(−0.016,0.011)	0.724	0.011(−0.007,0.030)	0.231	0.012(−0.010,0.035)	0.284
	Model 2	0.006(−0.006,0.018)	0.307	0.006(−0.006,0.017)	0.342	−0.003(−0.016,0.011)	0.680	0.014(−0.005,0.033)	0.231	0.020(−0.002,0.042)	0.081
PFUnDA	Model 1	−0.009(−0.021,0.004)	0.180	−0.003(−0.015,0.009)	0.596	0.001(−0.013,0.015)	0.900	−0.015(−0.035,0.004)	0.118	−0.019(−0.042,0.004)	0.112
	Model 2	−0.012(−0.024,0.0005)	0.059	−0.007(−0.018,0.005)	0.276	−0.001(−0.015,0.013)	0.879	−0.017(−0.037,0.002)	0.081	**−0.024(−0.047,0.001)**	**0.042**
PFHxS	Model 1	**0.014(0.0002,0.027)**	**0.047**	−0.003(−0.015,0.010)	0.692	**0.022(0.007,0.037)**	**0.004**	−0.015(−0.036,0.005)	0.149	−0.014(−0.038,0.010)	0.156
	Model 2	**0.015(0.002,0.028)**	**0.019**	−0.002(−0.014,0.011)	0.801	**0.022(0.008,0.037)**	**0.003**	−0.013(−0.033,0.008)	0.226	−0.014(−0.038,0.010)	0.250

PFOS	Model 1	0.003(−0.011,0.016)	0.707	0.004(−0.008,0.016)	0.580	−0.011(−0.026,0.004)	0.141	**0.023(0.002,0.043)**	**0.030**	**0.026(0.002,0.050)**	**0.034**
	Model 2	0.006(−0.007,0.019)	0.396	−0.007(−0.005,0.020)	0.241	−0.008(−0.023,0.006)	0.262	**0.023(0.003,0.044)**	**0.025**	**0.031(0.007,0.055)**	**0.013**
ΣPFAS	Model 1	0.005(−0.027,0.037)	0.751	−0.001(−0.031,0.029)	0.950	−0.004(−0.040,0.032)	0.826	0.014(−0.035,0.062)	0.579	0.013(−0.045,0.070)	0.664
	Model 2	0.004(−0.027,0.034)	0.815	−0.009(−0.021,0.038)	0.563	−0.004(−0.039,0.030)	0.809	0.014(−0.034,0.062)	0.571	0.023(−0.035,0.080)	0.437

Model1 is an unadjusted crude model, while Model2 adjusted for age, gender, marriage, family income, BMI, physical activity, sedentary time, smoking, drinking, hypertension and diabetes mellitus.

Table S3. Mediation effect of systemic inflammatory indicators in the association between PFAS exposure and NAFLD risk [a]

Parameters	Direct effect	Indirect effect	Total effect	Percentage mediated (%)	*p*-mediated
PFDA-NAFLD					
WBC	**0.041(0.019,0.064)*****	0.002(-0.003,0.007)	**0.043(0.020,0.067)*****	4.67	0.366
PLT	**0.043(0.018,0.067)*****	0.00004(-0.001,0.001)	**0.043(0.018,0.067)*****	0.02	0.942
LY	**0.044(0.022,0.066)*****	-0.001(-0.005,0.004)	**0.043(0.021,0.066)*****	-	-
NLR	**0.043(0.020,0.066)****	0.0001(-0.001,0.001)	**0.043(0.020,0.066)****	0.07	0.870
SII	**0.043(0.018,0.067)*****	0.0002(-0.0004,0.001)	**0.043(0.019,0.068) *****	0.32	0.568
PFOS-NAFLD					
WBC	**0.044(0.021,0.068)****	0.001(-0.004,0.006)	**0.045(0.021,0.070)****	2.20	0.664
PLT	**0.045(0.020,0.071)*****	0.0002(-0.001,0.001)	**0.045(0.020,0.071)*****	0.19	0.69
LY	**0.049(0.025,0.072)*****	-0.003(-0.008,0.001)	**0.045(0.021,0.069)****	-	-
NLR	**0.045(0.021,0.069)****	0.0001(-0.001,0.002)	**0.045(0.022,0.069)****	0.14	0.894
SII	**0.045(0.020,0.071) *****	0.0004(-0.001,0.002)	**0.045(0.020,0.071)*****	7.85	0.466
ΣPFAS-NAFLD					
WBC	**0.081(0.024,0.137)****	0.002(-0.009,0.014)	**0.083(0.025,0.141)****	2.46	0.712
PLT	**0.083(0.024,0.142)****	-0.0001(-0.002,0.002)	**0.083(0.023,0.142)****	-	-
LY	**0.084(0.026,0.140)****	-0.001(-0.011,0.009)	**0.083(0.024,0.140)****	-	-
NLR	**0.083(0.023,0.142)****	0.0001(-0.001,0.002)	**0.083(0.024,0.142)****	0.03	0.908
SII	**0.083(0.023,0.140)****	0.0002(-0.001,0.002)	**0.083(0.023,0.140)****	0.09	0.794

[a] The results are all crude models without any adjustments.

[b] The mediation ratio cannot be estimated because the direct and indirect effects are in opposite directions.

* $p < 0.05$, ** $p < 0.01$ and ***$p < 0.001$.

Table S4. Mediation effect of NAFLD in the association between PFAS exposure and systemic inflammatory indicators

Parameters	Direct effect	Indirect effect	Total effect	Percentage mediated (%)	*p*-mediated
Model1					
PFNA-NAFLD-LY	0.004(-0.008,0.016)	-0.001(-0.003,0.002)	0.004(-0.009,0.016)	-[a]	-
PFNA-NAFLD-NLR	**-0.020(-0.040,-0.002)***	-0.00003(-0.001,0.0004)	**-0.020(-0.039,0.002)***	0.03	0.890
PFUnDA-NAFLD-SII	-0.018(-0.042,0.004)	-0.0001(-0.001,0.0005)	-0.019(-0.042,0.004)	0.36	0.688
PFHxS-NAFLD-WBC	**0.015(0.002,0.028)***	-0.001(-0.003,0.001)	**0.014(0.0005,0.027)***	-	-
PFHxS-NAFLD-LY	**0.023(0.008,0.038)****	-0.001(-0.004,0.002)	**0.022(0.007,0.039)****	-	-
PFOS-NAFLD-NLR	**0.023(0.003,0.043)***	0.0001(-0.001,0.002)	**0.023(0.003,0.043)***	0.48	0.896
PFOS-NAFLD-SII	**0.026(0.002,0.049)***	0.001(-0.001,0.003)	**0.026(0.003,0.051)***	2.23	0.426
Model2					
PFNA-NAFLD-LY	0.004(-0.008,0.016)	-0.001(-0.003,0.001)	0.003(-0.009,0.015)	-	-
PFNA-NAFLD-NLR	**-0.020(-0.040,-0.001)***	-0.0002(-0.001,0.0003)	**-0.020(-0.040,0.001)***	0.68	0.530
PFUnDA-NAFLD-SII	-0.022(-0.046,0.002)	-0.0003(-0.002,0.001)	-0.022(-0.046,0.001)	1.09	0.634
PFHxS-NAFLD-WBC	**0.015(0.001,0.028)***	-0.001(-0.004,0.002)	**0.014(0.00004,0.028)***	-	-

PFHxS-NAFLD-LY	**0.022(0.008,0.037)*** **	-0.001(-0.003,0.001)	**0.022(0.006,0.036)*** **	-	-
PFOS-NAFLD-NLR	0.021(-0.0002,0.041)	0.001(-0.001,0.003)	**0.022(0.001,0.042)***	2.76	0.336
PFOS-NAFLD-SII	**0.027(0.002,0.051)***	0.002(-0.0003,0.004)	**0.029(0.005,0.053)***	4.46	0.160

Model 1 is an unadjusted crude model, while Model 2 adjusted for age, gender, marriage, family income, BMI, physical activity, sedentary time, smoking, drinking, hypertension and diabetes mellitus. [a] The mediation ratio cannot be estimated because the direct and indirect effects are in opposite directions.

* $p < 0.05$, ** $p < 0.01$ and *** $p < 0.001$.

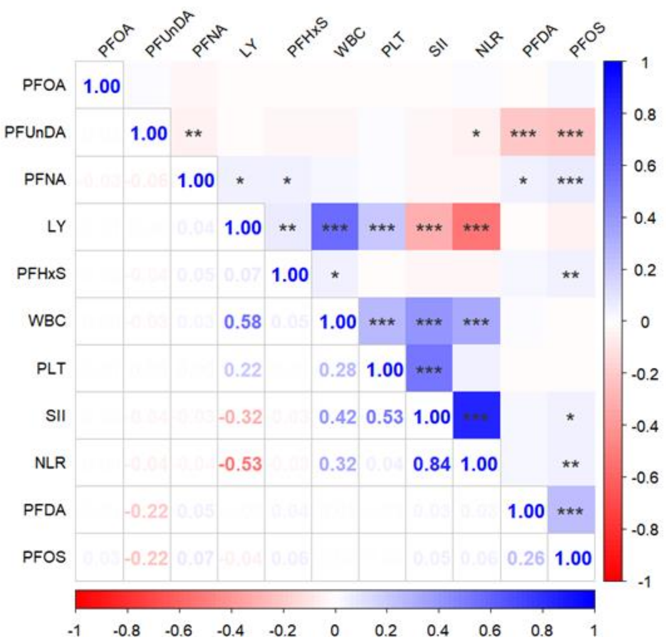

Figure S1. Spearman correlation matrix of PFAS and systemic inflammation indicators among the total population (n = 2191). The numbers in the figure represent the correlation coefficients; * $p < 0.05$, ** $p < 0.01$ and *** $p < 0.001$.
